# Toll-like receptors and NLRP3 inflammasome-dependent pathways in Parkinson’s disease: mechanisms and therapeutic implications

**DOI:** 10.1007/s00415-022-11491-3

**Published:** 2022-12-03

**Authors:** Luca Soraci, Maria Elsa Gambuzza, Leonardo Biscetti, Pasqualina Laganà, Carmela Lo Russo, Annamaria Buda, Giada Barresi, Andrea Corsonello, Fabrizia Lattanzio, Giuseppe Lorello, Gianfranco Filippelli, Silvia Marino

**Affiliations:** 1 Unit of Geriatric Medicine, Italian National Research Center on Aging (INRCA-IRCCS), 87100 Cosenza, Italy; 2 Territorial Office of Messina, Italian Ministry of Health, 98122 Messina, Italy; 3 Section of Neurology, Italian National Research Center on Aging (INRCA-IRCCS), 60121 Ancona, Italy; 4 Biomedical, Dental, Morphological and Functional Imaging Department, University of Messina, 98124 Messina, Italy; 5 Department of Clinical and Experimental Medicine, University of Messina, 98124 Messina, Italy; 6 Scientific Direction, Italian National Research Center on Aging (INRCA-IRCCS), 60121 Ancona, Italy; 7 Unit of Internal Medicine, Polyclinic G Martino Hospital, 98125 Messina, Italy; 8 Medical Oncology, Stabilimento Ospedaliero Paola, 87027 Cosenza, Italy; 9 IRCCS Centro Neurolesi Bonino-Pulejo, 98124 Messina, Italy

**Keywords:** Parkinson’s disease, Inflammation, Innate immunity, Toll-like receptors, α-synuclein, TLR/NLRP3/Caspase-1 pathway

## Abstract

Parkinson’s disease (PD) is a chronic progressive neurodegenerative disorder characterized by motor and non-motor disturbances as a result of a complex and not fully understood pathogenesis, probably including neuroinflammation, oxidative stress, and formation of alpha-synuclein (α-syn) aggregates. As age is the main risk factor for several neurodegenerative disorders including PD, progressive aging of the immune system leading to inflammaging and immunosenescence may contribute to neuroinflammation leading to PD onset and progression; abnormal α-syn aggregation in the context of immune dysfunction may favor activation of nucleotide-binding oligomerization domain-like receptor (NOD) family pyrin domain containing 3 (NLRP3) inflammasome within microglial cells through interaction with toll-like receptors (TLRs). This process would further lead to activation of Caspase (Cas)-1, and increased production of pro-inflammatory cytokines (PC), with subsequent impairment of mitochondria and damage to dopaminergic neurons. All these phenomena are mediated by the translocation of nuclear factor kappa-B (NF-κB) and enhanced by reactive oxygen species (ROS). To date, drugs to treat PD are mainly aimed at relieving clinical symptoms and there are no disease-modifying options to reverse or stop disease progression. This review outlines the role of the TLR/NLRP3/Cas-1 pathway in PD-related immune dysfunction, also focusing on specific therapeutic options that might be used since the early stages of the disease to counteract neuroinflammation and immune dysfunction.

## Introduction

Parkinson’s disease (PD) represents the second most common age-related neurodegenerative disease after Alzheimer’s disease (AD) in the elderly, with a raising social and economic burden on societies [1]. This disorder, affecting both the central nervous system (CNS) and the peripheral autonomic nerves, is mainly characterized by a progressive decline of nerve cells with consequent impairment of body movement, speech and frequently mental cognition [2]. The cardinal anatomo-pathological hallmarks are represented by the progressive loss of neuromelanin-containing dopaminergic neurons in the substantia nigra pars compacta (SNpc) [3], the presence of eosinophilic intracellular proteinaceous inclusions, termed Lewy bodies (LBs) [4], and Lewy neuritis [5].

Classically, the loss of SNpc neurons is seen as the cause leading to striatal dopamine (DA) deficiency, that is responsible for the major motor symptoms of PD [6]; however, PD pathology seems to start in other body areas, independently involving the parasympathetic neurons of intestinal plexus [7], olfactory bulb [8] and the lower brainstem [9], and spreading progressively from there to SNpc. Therefore, non-motor symptoms like hyposmia and constipation frequently precede the onset of motor dysfunction in PD [10].

In any case, despite PD pathogenesis is yet largely unknown, there are at least two main hypotheses to explain the onset and progression of the disease: the first posits that misfolding and aggregation of alpha-synuclein (α-syn) are pathologically linked to death of dopaminergic neurons, while the second hypothesis proposes that the culprit is mitochondrial dysfunction and the consequent oxidative stress, including generation of toxic oxidized DA species [11, 12].

Physiologically, the monomeric form of α-syn is abundant in mammals, and it seems to be involved in neuronal vesicle transport, transcriptional regulation, and modulation of immune cell function [13]. Despite α-syn misfolding in PD mainly affects neurons, recent data indicate that similar alterations may involve multiple CNS innate immune cell types, including astrocytes, oligodendrocytes and microglia [14, 15]. The innate immune response in the CNS is implicated in both beneficial and detrimental effects to health. Microglia, composed of the resident immune cells of the CNS, are considered “the brain macrophages”, able to shift from a surveillance mode to a reactive mode, so acting as immune effector cells producing pro-inflammatory cytokines (PC), and also contributing to cell-to-cell spread of misfolded α-syn protein between neurons, potentially leading to neurodegeneration and PD onset [16, 17]. Microglia, as well as other innate immune cells, express a wide variety of innate immune receptors, known as pattern-recognition receptors (PPRs), mainly including toll-like receptors (TLRs), and nucleotide-binding oligomerization domain-like receptors (NLRs) [18–20]. TLRs, which are also expressed on neurons and astrocytes, play a crucial role in inflammatory responses, also contributing to coordinate the activation of the adaptive immune system [20–22]. In PD, aggregated forms of α-syn were reported to activate microglia, through interaction with TLR2 and 4. This in turn causes the following activation of nucleotide-binding oligomerization domain-like receptor (NOD) family pyrin domain containing 3 (NLRP3) inflammasome mediates the Cas-1 activation and PC production, through the translocation of nuclear factor kappa-B (NF-κB). Finally, the release of PC may impair mitochondria and damage dopaminergic neurons [23]. This inflammatory cascade may be further exacerbated by mitochondrial dysfunction, that was early observed both in PD experimental models and in postmortem PD brain patients [24, 25]. Available evidence also suggests that neuroinflammatory mechanism observed in PD context, as well as in other neurodegenerative disturbances including Alzheimer’s disease, could be often favored by a large variety of immunological dysfunctions associated with age, now termed immunonosenescence [26].

Given the importance of inflammatory pathways in aging and PD pathogenesis [27], in this review, we aimed to update existing knowledge on the role of the α-syn/TLRs/NLRP3-Cas-1 inflammasome axis and microglial activation in PD [19, 27, 28], by exploring potential links between inflammaging and neurodegeneration; we also discussed advantages and limits of potential treatment options to modulate immune responses and counteract neuroinflammation in PD.

## Brain immunosenescence, neuroinflammation, and PD

Aging is one of the main risk factors for PD, since some neuroinflammatory mechanisms associated with aging also contribute to PD pathogenesis [29]. Indeed, aging is characterized by a complex process of immunosenescence, consisting in immunologic changes affecting both innate and acquired responses and associated with progressive immunodeficiency, chronic inflammation, decline in cellular clearance and autoimmunity [29, 30]. Dysregulation of senescent CNS immune cells was observed in both brain aging and PD progression [31]. Specifically, despite microglia physiologically recognize and remove extracellular α-syn aggregates originated from neuronal debris of apoptotic cells, the internalization of misfolded compounds may instead induce PC production, reduced nicotinamide adenine dinucleotide phosphate (NADPH) oxidase activation, and reactive oxygen species (ROS) generation, thus leading to phagocytic and clearance ability impairment [32, 33]. Together with microglial dysfunction, the increasing number of brain senescent immune cells also contributes to PC production and cell degeneration, potentially leading to PD development [34, 35].

From a general point of view, aging is associated with a state of chronic low-grade and multi-organ inflammation, that contributes to accumulation of unrepaired cellular damage, weakened cellular repair ability, and progressive immune dysregulation; this phenomenon, also known as “inflammaging” [34], is characterized by up-regulation of NF-κB signaling and cytokine/chemokine levels, inflammasome over-stimulation and lipid accumulation [36]. Therefore, inflammaging can be considered as a long-standing and self-perpetuating “pathogen-free inflammation”, which may contribute to PD pathogenesis [35, 37]; indeed, aged microglia are more responsive to pro-inflammatory stimuli inducing overexpression of several PC including NF-κB, and up-regulation of inflammasome pathways [38].

This link between aging and PD has been also supported by recent studies showing that an impaired proteasome/lysosome function, oxidative/nitrative damage, and inflammation processes on one hand increase with advancing age and, on the other hand, appear more evident in the ventral tier substantia nigra dopaminergic neurons, which are particularly vulnerable to PD-related degeneration [29, 39]. Furthermore, a dysregulation of microglial phagocytic activity, characterized by hyperactive microglia, with mixed pro-inflammatory and anti-inflammatory phenotypes, has been reported in PD brain [29, 39]. To this regard, it is noteworthy that aging is frequently associated with a dysregulation of physiological anti-inflammatory mechanisms, thus contributing to impairment of phagocytic mechanisms, as well as with increased sensitivity to stressors [40]. In PD, in addition to the above-mentioned aging-related immunological dysfunctions, the pro-inflammatory microglial profile promotes peripheral immune cells recruitment, and this further enhances neuroinflammation [40]. Moreover, the progressive genomic instability, typically associated to senescence processes, together with epigenetic alterations, and loss of protein homeostasis, might also contribute to dysregulation of innate immune responses associated to PD [41]. Recently, epigenetic mechanisms were shown to modulate neuroinflammation in PD, and several transcription factors appear to be master regulators of microglia reactivity [42]. Microglia and astrocyte activation involves TLR/Cas-1/NF-κB signaling pathway and leads to the release of PC, which further damage dopaminergic neurons, by inducing neuronal apoptosis and α-syn aggregation. Altogether, these synergic interactions form a vicious cycle that further exaggerates neuroinflammation [42]. The important role of neuroinflammation in PD progression is also confirmed by studies reporting that the inhibition of IFN-γ and TNF-α production by microglia and astrocytes can delay neuronal degeneration in PD animal models [43, 44].

Therefore, inflammation, senescence, and PD appear to be strictly related, and this is ultimately confirmed by recent data reporting that inflammatory and senescence markers share a similar profile, which seems to predict clinical progression in PD patients [45].

Moreover, age-associated inflammatory changes are characterized by enhanced activation of multiprotein complexes called inflammasomes [46]; multiple components of inflammasome, including NLRP3 and Cas-1, were found to be over-expressed in senile microglia mice [47], while higher NLRP3 expression levels have been detected both in age-related disease models [46] and in elderly subjects [48]. Enhanced activation of NLRP3 inflammasome associated with inflammaging [49] can cause overproduction of pro-inflammatory mediators, which in turn lead to synaptic plasticity degradation, and deleterious effects on neural precursor cells and normal neuronal functions [50–52]. The dysregulated NLRP3 function observed in aged mice confirms the involvement of enhanced expression of NLRP3 in cognitive dysfunction and motor performance, also suggesting that the abrogation of NLRP3 inflammasome can represent an innovative therapeutic target for multiple age-related neurological disorders [53, 54]. On the other hand, it was further shown that dopamine inhibits NLRP3 inflammasome activation, then preventing neuroinflammation [55]. Globally, these dysfunctions, mainly attributed to age-related changes, lead to abnormal protein accumulation in the brain, as particularly evidenced in the molecular pathogenesis of PD, where protein aggregation, mitochondrial dysfunction, together with inflammation, have been shown to coexist [56].

## TLRs in aging and PD

Classically, CNS has been regarded as an “immunologically privileged site”, because the blood–brain barrier (BBB) was believed to prevent many molecules, including antibodies, from crossing over into the CNS and the brain was considered to be devoid of macrophages and lymphocytes. However, recently a growing body of evidence indicates that innate immunity-related molecules, including cytokines, TLRs, the complement family, and acquired immunity-related mediators are also expressed in the brain [57, 58].

Neurons and innate immune cells express a wide variety of immune receptors among which the TLRs play an important role in inflammatory responses [18]. All eleven germ line-encoded human TLRs consist of two domains joined by a single transmembrane helix and form homodimers or heterodimers, as a means of triggering a signal cascade, resulting in activation of the responding cell [59]. TLR1, TLR2, TLR4, TLR5, TLR6, TLR10 reside in the plasma membrane and recognize extracellular pathogens and endogenous ligands released from damaged tissues [20]. In contrast, the TLR3, TLR7, TLR8, TLR9, and TLR11 are localized in intracellular organelles and recognize patterns of DNA or RNA, or endogenous nucleic acids released by necrotic or late apoptotic cells and host derived peptides [60]. Activation of neuronal and microglial TLRs during normal aging might constitute a possible link between inflammaging and many neurodegenerative diseases, including PD [18].

### Role of TLRs in synucleinopathies

Increasing evidence indicates that α-syn interacts with both TLR2 and TLR4 to mediate immune activation allowing α-syn aggregation and chronic inflammation [61], thus leading to progressive damage to neuronal cells; furthermore, chronic activation of gut and enteric cell TLRs secondary to microbial dysbiosis may further contribute to impaired immunity and disease progression in PD patients [62].

Among the heterogeneous family of TLRs, TLR2 and TLR4 seem to represent crucial regulator of inflammation in PD synucleinopathy, since elevated α-syn alone is not sufficient to cause PD [34, 61]. Specifically, both TLR2 homodimers and TLR2/TLR1 and TLR2/TLR6 heterodimers have been shown to bind directly the fibrillary α-syn, triggering TNF and IL-1β production [34]. On the other hand, TLR4 interaction with α-syn appears to mediate its uptake, promoting a pro-inflammatory status characterized by cytokine production and ROS generation by both microglia and astroglia [34]. In any case, the prolonged TLR-mediated inflammation may trigger α-syn misfolding into oligomers and fibrils, which in turn interacts with TLR2 and/or TLR4 in a vicious circle, and negatively affect other PD-related mechanisms, including proteasome induction and mitochondrial dysfunction [34]. TLR2 and TLR4 stimulation by α-syn can also trigger NF-κB-dependent PC production and up-regulate NLRP3 component of the inflammasome, thus further promoting neuroinflammation and contributing to PD progression [63]. The α-syn aggregates engulfed by the microglia induce damage of lysosomes and their leaking into the cytoplasm, with further contribution to inflammasome activation. Furthermore, recent evidence shows that TLR9 up-regulation in human striatal homogenates might contribute to PD neurodegeneration, by activating an inflammatory pathway regulated by glucocorticoids [64].

## NLRP3 pathway and mitochondrial dysfunction

### Role of NLRP3 inflammasome in aging and PD

**Fig. 1:**
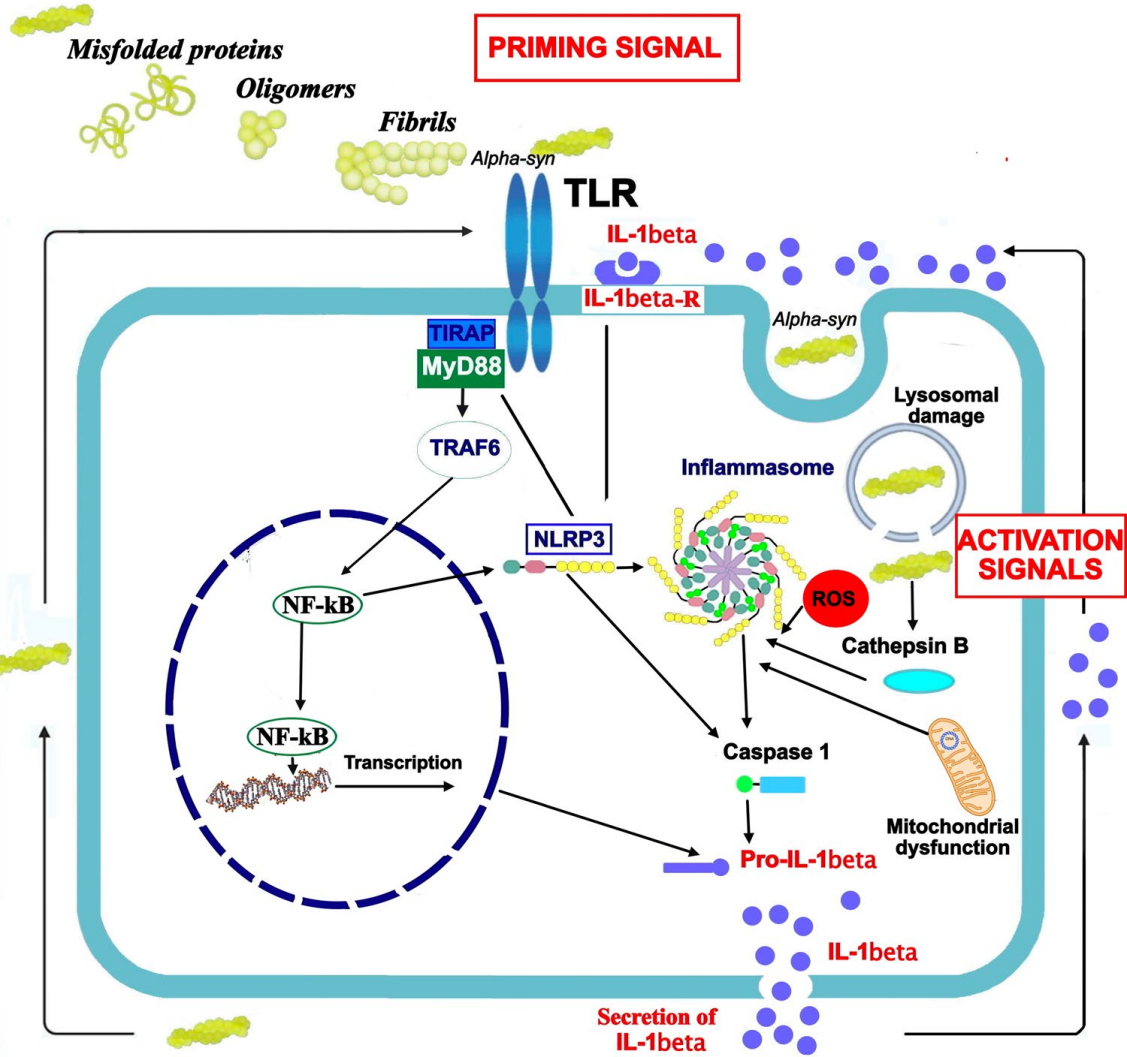
A two-signal model for NLRP3 inflammasome activation. A bimodal signaling pathway is required to induce the NLRP3 inflammasome activation: the TLR-dependent priming signal (signal 1) is provided by alpha-syn or endogenous cytokines, and leads to the activation of the transcription factor NF-kB and subsequent up-regulation of NLRP3 and pro-interleukin-1β (pro-IL-1β); NLRP3 undergoes post-translational modifications that license its activation. The activation signal (signal 2), provided by a variety of stimuli and multiple molecular or cellular events, including ionic flux, mitochondrial dysfunction with ROS generation, and lysosomal damage, activates the NLRP3 inflammasome, with subsequent activation (cleavage) of Caspase-1, that in turn catalyzes the cleavage of IL-1β and IL-18. The NLRP3 inflammasome may also be activated by agents that cause mitochondrial dysfunction, leading to generation of mitochondrial ROS. *IL-1beta-R* IL-1β receptor, *TLR* toll-like receptor, *alpha-syn* alpha-synuclein, *ROS* reactive oxygen species

TLR- and α -synuclein-induced activation of microglial NLRP3 inflammasomes may contribute to PD progression [63]. Inflammasomes consist of multimeric protein complexes involved in the initiation and propagation of immune responses [65]. The Nod-like receptor (NLR) family pyrin domain containing 3 (NLRP3) inflammasome is a cytoplasmatic complex involved in the production of IL-1β and able to induce pyroptosis, a fast inflammatory form of lytic programmed cell death [66]. NLRP3 inflammasome is composed by a sensor protein (NLRP3), an adaptor component (ASC or PYARD), and an effector (caspase 1) [66]; its activation is a tightly regulated process which occurs in response to various inflammatory stimuli, including bacteria, viruses and cellular components [65]. A successful activation of the NLRP3 inflammasome depends from two signals: priming signals, able to mediate the transcription of NLRP3 and pro-IL-1β and pro-IL-18; activation signals able to promote assembly and activation of the inflammasome complex [23]. Priming signals are mainly represented by damage-associated molecular patterns (DAMPs) and TLR ligands, as well as IL-1α; activation signals include adenosine triphosphate (ATP), viral DNA, and misfolded proteins [23]. The NLRP3 inflammasome plays a pivotal role in guiding host immune responses against bacterial, viral, and fungal infections [67]; however, its dysregulated activation was associated with the onset and progression of several age-related pro-inflammatory diseases, such as diabetes, atherosclerosis, gout, as well as neurodegenerative disorders, like Alzheimer’s disease and PD [65]. Activation of NLRP3 in PD is a two-step process (Fig. 1).

The TLR-dependent priming signal (signal 1) is provided by alpha-syn or endogenous cytokines, and leads to the activation of the transcription factor NF-kB and subsequent up-regulation of NLRP3 and pro-interleukin-1β (pro-IL-1β) [68]; NLRP3 undergoes post-translational modifications that license its activation. The activation signal (signal 2), provided by a variety of stimuli and multiple molecular or cellular events, including ionic flux, mitochondrial dysfunction with ROS generation, and lysosomal damage, activates the NLRP3 inflammasome, with subsequent activation (cleavage) of Caspase-1, that in turn catalyzes the cleavage of IL-1β and IL-18.

### Interplay between NLPR3 inflammasome and mitochondrial dysfunction

The NLRP3 inflammasome can also be activated by agents that cause mitochondrial dysfunction, leading to generation of mitochondrial ROS [68]. Available evidence suggests that there is a synergistic relationship between mitochondrial dysfunction and the mechanism of NLRP3 activation in PD pathogenesis. Indeed, on one hand, mitochondrial dysfunction may increase the activity of the NLRP3 inflammasomes [69]; on the other hand NLRP3 contributes to mitochondrial impairment, thus promoting mitochondrial DNA (mtDNA) and cytochrome c release in cytosol [70]. Moreover, the production of mitochondrial reactive oxygen species (mtROS) and dysregulated mitophagy (i.e., a particular form of autophagy leading to a selective degradation of mitochondria) are the key regulators of NLRP3 activation [71]. Based on this, a recent investigation reported that mitophagy may inhibit the activation of NLRP3 in microglia in a PD model, thereby reducing inflammation and improving neuronal loss [72]. Another study reported that cardiolipin, a mitochondria-specific phospholipid located physiologically in the inner membrane of mitochondria, after translocation to the outer membrane induces the assembly of NLRP3, again supporting the link between mitochondria impairment and inflammasome [73]. Of note, rotenone, a potent pesticide associated with idiopathic PD, acts as mitochondria poison, inhibiting complex I of the mitochondrial electron transport chain, thus resulting in mtROS production, mitochondrial dysfunction, and NLRP3 signaling [74]. Other drugs with detrimental effects on mitochondria function including imiquimod, also activate NLRP3 signaling via oxidative stress [75]. Taken together, these data suggest the opportunity to deeply investigate the intriguing crosstalk among mitophagy, oxidative stress, neuroinflammation and neurodegeneration in PD pathophysiology to develop new disease-modifying strategies.

Altogether, the immune response to protein accumulation appears to trigger deleterious events, such as oxidative stress and cytokine receptor-mediated cell death, which lead to neuronal loss. Whereas the activation of glia can play a neuroprotective role in the first stage of the disease, the chronic immune activation can lead to a closed circuit of auto sustaining inflammation, involving also T-cell infiltration from the periphery, that may favor disease progression.

## Therapeutic targeting of PD-related inflammatory pathways

Current approaches for PD aim to reduce motor impairment by both maintaining the normal DA levels and inhibiting the degradation of endogenous DA, in addition to supply the DA precursor levodopa or DA agonists. However, these therapeutic approaches show only limited effectiveness in ameliorating clinical symptoms and have been shown to have untoward side effects, like motor fluctuations, and dyskinesias [76].

Anyway, to date, there are no current disease-modifying therapies for PD patients and developing safer and more effective therapies is an immediate and important challenge. In this view, since the neuroinflammation plays a crucial role in PD progression, immunomodulatory therapies may represent a promising approach in PD treatment. A specific modulation of TLRs/NLRP3/Cas-1 inflammasome axis and microglial activation might represent a more effective treatment. More specifically, a very promising immunotherapeutic intervention could be obtained using specific modulators TLR-targeting yet indicated for the treatment of other inflammatory diseases, in addition to NLPR3 and Cas-1 modulators.

### TLR-modulatory options

TLR modulation by specific antagonists could represent effective therapeutic strategy to treat or prevent both systemic inflammatory diseases and neuroinflammatory disorders. TLR antagonists are molecules able to downregulate activation of TLR-mediated cytokine production, by directly binding to specific TLRs, or indirectly blocking pro-inflammatory signaling cascade [77, 78]. TLR2 and TLR4 are currently being investigated as potential therapeutic targets in inflammatory diseases, including sepsis and arthritis, as well as neurodegenerative disorders [79, 80]. Three major types of PD models have been used to investigate potential benefits of TLR-modulatory compounds in PD: human subjects with PD, animal models, and/or cultured microglial cells (Table 1).

**Table 1:** The main TLR-modulatory compounds carrying in vitro and/or in vivo activity to counteract inflammation in PD models

Name	Activity	Model/Study	Effects
Vinpocetine	Decreases mRNA levels of TLR2, TLR4, MyD88, and NF-κB	Patients with PD, double-blind placebo control study [81]	Decreased serum levels of pro-inflammatory cytokines, induced TLR3 and serum anti-inflammatory substances. Not clear the effects on cognitive function
Functional antibody	TLR2 antagonist	Murine PD model [82]	Decreased α-syn aggregation and deposition, lowered neuroinflammation, neurodegeneration and behavioral symptoms
Farrerol	TLR4 inhibitor	Murine PD model [83]	Decreased production of pro-inflammatory cytokines, including TNF-α, IL-6, and IL-1β; increased inhibition of NF-κB
Kaempferol	Down-regulation of TLR4-mediated pathway	Murine PD model [84]	Decreased production of pro-inflammatory cytokines, including TNF-α, IL-6, and IL-1β; decreased neuronal injury
Hesperetin	Down-regulation of TLR4-mediated pathway	Murine PD model [85]	Improved cognition, memory and synaptic plasticity
Candesartan	Antihypertensive medication decreasing TLR2 expression	Cultured primary microglia [86]	Shifted primary microglia status from pro-inflammatory to anti-inflammatory
CU-CPT22	TLR1/2 heterodimer inhibitor	Cultured primary microglia [86]	Decreased production of pro-inflammatory cytokines, including TNF-α and IL-1β; decreased translocation of NF-κB
Schisandrin B	Inhibitor of interaction between TLR4 and regulatory proteins (MyD88, TRAF-6)	Cultured microglia; murine PD model [87]	Decreased production of pro-inflammatory cytokines, including TNF-α, IL-6, PGE2, and IL-1β, and ROS; inhibition of NADPH oxidase
Dihydrotestosterone (DHT)	Inhibitor of TLR4-dependent NF-kB and MAPK p38 pathways	Cultured microglia; murine PD model [88]	Decreased production of pro-inflammatory cytokines, improvement of neurocognitive tests
Silymarin	Suppresses TLR4-dependent pathway	Murine PD model [89]	Neuroprotective effects on dopaminergic neurons, by reducing neuronal apoptosis
Icariside II	Inhibitor of TLR4/MyD88/NF-kB pathway	Murine PD model [90]	Decreases pro-inflammatory cytokines production, and astrocyte activation [85–87]
Fecal microbiota transplantation (FMT)	Among others, decreases TLR4 gut and brain expression	Murine PD model [91]	Increase in striatal dopamine, reversal of microglial and astrocyte activation, decreased TLR4 gut and brain expression

To date, vinpocetine, a semisynthetic derivative of alkaloid vincamine, results to be the only TLR-modulatory compound tested in PD patients within a small randomized trial [81]. According to this study, vinpocetine, decreases mRNA levels of TLR2, TLR4, MyD88, and NF-κB compared to standard therapy. However, the clinical impact of this finding is still unclear and further studies will be necessary to clarify the usefulness of vinpocetine for PD treatment.

Other compounds showing some benefit in reducing neuroinflammation were tested in animal and cellular PD models [19], (Table 1). Among them, a functional antibody capable of inhibiting TLR2 in PD mouse models substantially decreased aggregation and deposition of α-synuclein within neurons and microglia as well as neuroinflammation and neurodegeneration [82]; similarly, the small molecule CU-CPT22 inhibits the heterodimer TLR1/TLR2 and showed to have some neuroprotective and anti-inflammatory actions in cultured microglial cells [86]. Furthermore, the antihypertensive medication candesartan cilexetil can reverse the activated pro-inflammatory status of cultured microglial cells exposed to α-synuclein, by decreasing TLR2 expression [86]. Many natural compounds including farrerol, kaempferol, dihydrotestosterone, silymarin, and hesperidin showed anti-inflammatory effects mediated by inhibition of TLR4 or TLR4-dependent pathways, further decreasing the production of pro-inflammatory cytokines in cellular and murine PD models [82–84, 88, 90]; the flavonoid silymarin, in particular, has been shown neuroprotective effects by reducing neuronal apoptosis, through inhibition of TLR4 over expression, in dopaminergic neurons in murine SNpc [89].

Another promising therapeutic approach consists in the fecal microbiota transplantation (FMT), as recent evidence supports the involvement of the gut–brain axis in the onset and progression of PD [91]. Use of FMT in PD animal models was associated with increased dopamine production in the striatum, reversal of microglial and astrocyte activation, as well as decreased gut and brain TLR4 expression [92]; additionally, studies involving a small number of individuals with PD have shown FMT potential in decreasing non-motor symptoms [93, 94].

### NLRP3/Cas-1 modulators

#### Data on molecules tested in PD field

In the last years, numerous studies have been performed to discover innovative therapeutic strategies aimed to fight neurodegenerative diseases through the inhibition of the NLRP3/Cas-1 inflammasome pathway [48]. Among the endogenous mechanisms of inflammasome regulation, dopamine has been shown to play an important role to control systemic inflammation, by acting as endogenous inhibitor of the NLRP3 inflammasome pathway [55]. NLRP3/Cas-1 modulators could selectively suppress inflammation caused by the NLRP3 inflammasome, either by directly targeting NLRP3 and NLRP3-dependent pathways (NF-kB pathway and ROS synthesis) or by inhibiting regulatory proteins involved in PD neurodegeneration, such as caspase-1 [67]. However, there are yet no clinically approved compounds for targeting of NLRP3 or Cas-1 [66], but several molecules have shown promising results in cellular and murine PD models (Table 2).

**Table 2:** The main NLRP3/cas-1 inhibitors to counteract inflammation in PD models

Name	Activity	Model/Study	Effects
Direct NLRP3 inhibitors			
MCC950 (CP-456,773)	Small molecule NLRP3 inhibitor	Cultured microglia; Murine PD model [95]	Neuroprotective effects on dopaminergic nigrostriatal neurons; inhibition of release of IL-1β and caspase 1
Kaempferol	NLRP3 inhibitor	Murine PD model [96]	Decreases neuronal apoptosis and production of pro-inflammatory cytokines; increases mRNA and protein expression of tyrosine hydroxylase
Oridonin	NLRP3 inhibitor	Cultured cells [97]	Decreases production of ROS and pro-inflammatory cytokines
Fingolimod (FTY-720)	NLRP3 inhibitor	Cultured microglia; Murine PD model [98]	Decreases dopaminergic neurodegeneration
AZ11645373	NLRP3 inhibitor, P2X7 receptor antagonist	Murine PD model [99]	Inhibits release of IL-1β
Celastrol	NLRP3 inhibitor	Murine PD model [100]	Relieves motor deficits and nigrostriatal dopamine degeneration
Inhibitors of NLRP3-mediated pathways			
Dapagliflozin	NF-κB pathway inhibitor	Murine PD model [101]	Suppresses neuroinflammation by decreasing ROS production; decreases TNF-α levels
Lenalidomide	NF-κB pathway inhibitor	Murine PD model [102, 103]	Decreased pro-inflammatory cytokine production; suppresses levels of phosphorylated NF-κB; reduces microglial activation; improves motor and behavioral symptoms
Triptolide	NF-κB pathway inhibitor (via miRNA 155-5p/SHIP1 pathway)	Murine PD model [104]	Decreases microglial activation and production of pro-inflammatory cytokines
Juglanin	TLR4 and NF-κB pathway inhibitor	Murine PD model [105]	Decreases production of IL-1β, IL-18, TNF-α, and COX-2
Calycosin	TLR/NF-κB and MAPK pathways inhibitor	Cultured microglial cells; murine PD model [106, 107]	Decreases production of pro-inflammatory cytokines; alleviates behavioral symptoms
Diosgenin	TLR/NF-κB pathway inhibitor	Cultured microglial cells; murine PD model [108]	Decreases ROS production; decreases expression of TLR2, TLR4 and NF-κB; decreases mRNA levels of pro-inflammatory cytokines
Isobavachalcone	NF-κB pathway inhibitor	Cultured microglial cells; murine PD model [109]	Decreases production of pro-inflammatory cytokines and microglial activation
Apocynin	NADPH oxidase inhibitor	Murine PD model [110]	Decreases ROS production; prevents learning deficits
Diphenyleneiodonium	NADPH oxidase inhibitor	Murine PD model [111]	Decreases expression of pro-inflammatory cytokine and ROS genes
miRNA-7	NLRP3 inhibitor	Cultured neuronal and microglial cells; murine PD model [112]	Neuroprotective effects on dopaminergic neurons; inhibition of NLRP3 activation
miRNA-30e	NLRP3 inhibitor	Cultured neuronal and microglial cells; murine PD model [113]	Inhibits mRNA and protein NLRP3 synthesis; decreases production of pro-inflammatory cytokines
Caspase-1 inhibitors			
Necrostatins	Cas-1 inhibitor	Cultured microglial cells [114]	Increases neuroprotection on dopaminergic neurons

Among direct NLRP3 inhibitors, the MCC950 (or CP-456,773), is a very potent compound able to prevent inflammation and dopaminergic death in PD murine models [95]. Other potentially useful direct NLRP3 antagonists are represented by microRNA-153, microRNA-223 and microRNA-30e, whose plasma levels resulted to be decreased in PD [113, 115]. Additionally, microRNA-30e is a negative NLRP3 regulator and its administration exerts neuroprotective effects on murine models with PD, by decreasing the loss of dopaminergic neurons and improving motor and behavioral symptoms [113]. Other ways of targeting NLPR3 inflammasome are represented by inhibition of NF-kB pathway and ROS synthesis, which are both necessary for NLPR3 assembly and activation (Table 2). At this regard, recent data indicated that dapagliflozin, a sodium–glucose cotransporter 2 used for treatment of diabetes mellitus and heart failure, may alleviate neuronal oxidative stress by counteracting ROS production and NF-kB pathway activation in animal PD models [101]. Alternatively, use of inhibitors of regulatory proteins including caspases might be of some benefit in PD treatment. For instance, necrostatins are a family of Cas-1 inhibitors able to block necrotic cell death in human and murine cells exerting neuroprotective effects on dopaminergic neurons in murine PD models [114].

#### Data on promising NLRP3/Cas-1 modulators not yet tested in PD field

Beyond the above-mentioned NLRP3 antagonists already investigated in PD field (at least in pre-clinical studies), other drugs able to inhibit NLRP3 inflammasome with proved good pharmacokinetic profiles and safety and already used for other diseases, might be proposed for clinical trials in PD. Furthermore, in recent studies disulfiram, belonging to “anti-abuse drugs” and used to treat alcohol dependence, has been shown to inhibit both NLRP3 inflammasome activation and Gasdermin D-mediated pyroptosis, by specifically blocking pore formation, and IL-1β release [116].

In addition, several flavonoids have been found to affect the inflammasome pathway, among which, in particular, Flavonoid VI-16 has been reported to inhibit, both “in vitro” and “in vivo” experiments, the expression of IL-1 β, IL-18, and Cas-1, through inhibition of NLRP3 assembly [117].

Parthenolide, the first natural product that directly targets Cas-1 and NLRP3, has recently shown versatile inhibitory actions in different pathologies, such as AD [118]; similarly, ibrutinib, an FDA-approved natural products for the treatment of chronic lymphocytic leukemia and mantle cell lymphoma, has shown potential effects in preventing and reducing neuroinflammatory symptoms of AD, by targeting NLRP3/Cas-1 signaling [118].

## Discussion

An increased quantum of studies has greatly improved our knowledge on TLRs and inflammasomes and their role played both in physiological and pathological states, including neurological diseases. Today, there is yet no disease-modifying drug for PD, and the current pharmacological and non-pharmacological options do not address the underlying disease and do not stop or delay the cell damage that eventually leads to worsening of symptoms. In PD, both α-syn-clearance and inflammation are linked to TLR and inflammasome activation, which in turn lead to neuronal loss. Activation of specific TLRs promotes α-syn-clearance in the early stage, but the same TLRs, chronically activated by accumulated α-syn, induce a pro-inflammatory cascade, leading to degenerative changes to neurons during the middle/late stage of disease processes. In addition, numerous evidences show that in PD patients, systemic inflammation, mainly associated with microglia activation, can further enhance the DA neuron degeneration [119]. To this regard, disruption of the brain–gut axis secondary to intestinal microbial dysbiosis is recently emerging as a contributing factor in PD pathogenesis [62]; alteration of gut microbiota and gut epithelial barrier induces activation of enteric TLRs, thus promoting inflammation and oxidative stress in both gut and brain regions.

Therapeutic approaches in PD patients could be different. In this regard, use of TLRs/NLRP3 pathway modulators could be proposed as an alternative or complementary approach to L-DOPA administration with the aim of decreasing the burden of neuroinflammation from the earliest stages of the disease. To date, L-DOPA is the gold-standard treatment for PD, but its administration usually starts at a late stage, after the onset of symptoms, to limit the risk of iatrogenic dyskinesia secondary to its long-term use [120]; however, it is likely that activation of TLRs and NRLP3-mediated pathways starts years before the clinical onset of the disease; for this reason, despite the relationship between use of TLRs/NLRP3 modulators and clinical PD stage has not yet been investigated, we can speculate that use of TLRs and NLRP3 pathway modulators could be key to preventing harms related to neuroinflammatory mechanisms leading to loss of dopamine from the earliest stages of the disease.

Accordingly, early diagnosis and monitoring of treatment efficacy are essential. Researchers are focusing on identifying measurable biomarkers, associated with both CNS and peripheral inflammation, for early diagnosis, before the disease has caused an irreversible damage to brain cells, in order to allow clinicians to start the most effective treatments at the stage known as “pre-clinical PD” [121]. Therefore, both the progress in making an early diagnosis and the current knowledge on the role of autophagy and inflammation in PD progression may allow to select specific immunomodulators. Many of these drugs are currently used in clinical routine or tested in clinical trial for other pathologies and in the early future they might be studied also in PD field to try to improve the treatment of this disease.

A major problem to be overcome in drug design is linked to the ability of the selected compounds to cross the BBB. Substances can cross the BBB by a variety of mechanisms, including transmembrane diffusion, saturable transporters, adsorptive endocytosis, and the extracellular pathways. Generally, only lipid-soluble molecules with a molecular weight under 400–600 Da and positive charge can cross the BBB, whereas other molecules do not pass or can overcome the BBB only through specific cell endogenous transport systems [122]. In addition, the optimum size of small neuroactive drugs administered by peripheral infusion for their delivery into the brain is determined by competition between BBB permeation and excretion from blood circulation. Despite small particles are better for permeating through gaps opened in the BBB, smaller particles in the single-nm range are rapidly excreted from blood circulation via renal clearance [123]. Consequently, both antibodies and specific immunomodulators able to counteract peripheral inflammation, and which target other parts of the body, do not normally cross the BBB to the human brain. Among these, anti-TLR2 and -TLR4 neutralizing antibodies may be considered an interesting approach to block peripheral TLR-mediated inflammation, despite they have no inhibitory effect on brain inflammation. In any case, the task of predicting the BBB permeability of new compounds is a major challenge. Among the small compounds able to cross the BBB, there are small antigen-binding fragments, consisting in single-domain antibodies, and also known as nanobodies. Some TLR-specific nanobodies are capable of stimulating or inhibiting TLRs expressed by microglia, then exerting their direct effects on CNS and representing a promising approach to treat a range of serious and life-threatening human diseases, including neuro inflammatory, thrombotic, neoplastic, and neurodegenerative disorders, including AD and PD [124]. Nanobodies exhibit high affinity, have the potential to be administered to patients as inhaled drugs, skin patches or pills, and this easy regular administration also allows a successful combination therapy, depending on the PD stage. Tailored half-life formats allow molecule to remain in circulation for days, ideally customized, according to need. Among the innovative nanobody-based agents, there are specific intrabodies, able to cross cell membrane, bind intracellular α-syn monomers and block their oligomerization [125]. In particular, two proteasome-directed nanobodies, selectively targeting α-syn, were shown to restore striatal DA tone and enhance motor function in the α-syn-based PD model [125]

Since NLRP3 inflammasome appears to be a key molecular link in the PD inflammatory pathway, targeting selectively NLRP3/Cas-1 pathway with small molecule inhibitors can represent a valid approach for treating neuroinflammatory diseases. Many of these compounds, already analyzed “in vitro” and “in vivo”, have not yet verified by clinical trials for their ability to cross the BBB, the safety profile and therapeutic effects. Therefore, a large amount of work is still needed to be put in for the development of these inhibitors until they become gold-standard drugs capable of helping in reducing the social burden of the disease and improving the patients’ quality of life. Innovative nanotechnology methods have recently applied to resolve some general problems affecting these immunomodulators, including insufficient stability, poor water solubility, injection site aggregation, systemic toxic effects, not lasting effect, together a nonspecific immune suppression [126]. According to a recent study, exosome-like nanoparticles from ginger rhizomes (G-ELNs) were able to strongly inhibit NLRP3 inflammasome activation [127]; similarly, lipid/peptide nanoparticle emulsions were shown to block NLRP3 inflammasome activation by decreasing plasma LDH, potassium and chloride ions [128]; furthermore, garlic chive-derived vesicle-like nanoparticles (GC-VLNs) were found to have a potent inhibitory effect on NLRP3 downstream pathways, thus showing potential for treatment of neuroinflammatory diseases [129].

Developing accurate targeted drugs and effective delivery methods are another important issue, and the application of innovative biomaterials, and drug delivery devices, main represented by nanocarriers, may address these problems. Polymer nanoparticles are able to mediate passive or active targeted drug transport, improving both the drug concentration at the target organs, and the stability of loading drugs. By changing the size of the polymer nanoparticles, the clearance of small drug molecules from the kidney or liver can be reduced, thereby increasing the drug cycle time [130]. We look forward to the exciting progress of nanotechnology sciences and basic biology, together with the growing knowledge concerning the role of specific innate immune receptors and inflammation in PD and the translational studies of TLRs and NLRP3/Cas-1 inhibitors.

## Data Availability

The authors take full responsibility for the data, the analysis, and interpretation of the research, and they have full access to all of the data.

## References

[1] Aarsland D, Batzu L, Halliday GM, Geurtsen GJ, Ballard C, Ray Chaudhuri K, Weintraub D (2021). Parkinson disease-associated cognitive impairment. Nat Rev Dis Primers.

[2] Borghammer P, Knudsen K, Brooks D (2016). Imaging systemic dysfunction in Parkinson’s disease. Curr Neurol Neurosci Rep.

[3] Isaias IU, Trujillo P, Summers P, Marotta G, Mainardi L, Pezzoli G, Zecca L, Costa A (2016). Neuromelanin imaging and dopaminergic loss in Parkinson's disease. Front Aging Neurosci.

[4] Menšíková K, Matěj R, Colosimo C, Rosales R, Tučková L, Ehrmann J, Hraboš D, Kolaříková K, Vodička R, Vrtěl R (2022). Lewy body disease or diseases with Lewy bodies?. npj Parkinson's Dis.

[5] Braak H, Sandmann-Keil D, Gai W, Braak E (1999). Extensive axonal Lewy neurites in Parkinson's disease: a novel pathological feature revealed by alpha-synuclein immunocytochemistry. Neurosci Lett.

[6] Gordián-Vélez WJ, Chouhan D, España RA, Chen HI, Burdick JA, Duda JE, Cullen DK (2021). Restoring lost nigrostriatal fibers in Parkinson's disease based on clinically-inspired design criteria. Brain Res Bull.

[7] Hawkes CH, Del Tredici K, Braak H (2007). Parkinson's disease: a dual-hit hypothesis. Neuropathol Appl Neurobiol.

[8] Chen F, Liu W, Liu P, Wang Z, Zhou Y, Liu X, Li A (2021). α-Synuclein aggregation in the olfactory bulb induces olfactory deficits by perturbing granule cells and granular–mitral synaptic transmission. npj Parkinson's Dis.

[9] Uemura N, Ueda J, Yoshihara T, Ikuno M, Uemura MT, Yamakado H, Asano M, Trojanowski JQ, Takahashi R (2021). α-synuclein spread from olfactory bulb causes hyposmia, anxiety, and memory loss in BAC-SNCA mice. Mov Disord.

[10] De Rui M, Inelmen EM, Trevisan C, Pigozzo S, Manzato E, Sergi G (2020). Parkinson's disease and the non-motor symptoms: hyposmia, weight loss, osteosarcopenia. Aging Clin Exp Res.

[11] Guo JD, Zhao X, Li Y, Li GR, Liu XL (2018). Damage to dopaminergic neurons by oxidative stress in Parkinson's disease (Review). Int J Mol Med.

[12] Kaur I, Behl T, Sehgal A, Singh S, Sharma N, Aleya L, Bungau S (2021). Connecting the dots between mitochondrial dysfunction and Parkinson’s disorder: focus mitochondria-targeting therapeutic paradigm in mitigating the disease severity. Environ Sci Pollut Res.

[13] Kasen A, Houck C, Burmeister AR, Sha Q, Brundin L, Brundin P (2022). Upregulation of α-synuclein following immune activation: Possible trigger of Parkinson's disease. Neurobiol Dis.

[14] Haenseler W, Zambon F, Lee H, Vowles J, Rinaldi F, Duggal G, Houlden H, Gwinn K, Wray S, Luk KC (2017). Excess α-synuclein compromises phagocytosis in iPSC-derived macrophages. Sci Rep.

[15] Reynolds RH, Botía J, Nalls MA, Noyce AJ, Nicolas A, Cookson MR, Bandres-Ciga S, Gibbs JR, Hernandez DG, Singleton AB (2019). Moving beyond neurons: the role of cell type-specific gene regulation in Parkinson’s disease heritability. npj Parkinson's Dis.

[16] Booms A, Coetzee GA (2021). Functions of intracellular alpha-synuclein in microglia: implications for Parkinson's disease risk. Front Cell Neurosci.

[17] Qin Y, Qiu J, Wang P, Liu J, Zhao Y, Jiang F, Lou H (2021). Impaired autophagy in microglia aggravates dopaminergic neurodegeneration by regulating NLRP3 inflammasome activation in experimental models of Parkinson's disease. Brain Behav Immun.

[18] Fiebich BL, Batista CRA, Saliba SW, Yousif NM, de Oliveira ACP (2018). Role of microglia TLRs in neurodegeneration. Front Cell Neurosci.

[19] Kouli A, Horne CB, Williams-Gray CH (2019). Toll-like receptors and their therapeutic potential in Parkinson's disease and α-synucleinopathies. Brain Behav Immun.

[20] Pascual M, Calvo-Rodriguez M, Núñez L, Villalobos C, Ureña J, Guerri C (2021). Toll-like receptors in neuroinflammation, neurodegeneration, and alcohol-induced brain damage. IUBMB Life.

[21] Kawai T, Akira S (2010). The role of pattern-recognition receptors in innate immunity: update on Toll-like receptors. Nat Immunol.

[22] Satoh T, Akira S (2016). Toll-like receptor signaling and its inducible proteins. Microbiol Spectr.

[23] Kelley N, Jeltema D, Duan Y, He Y (2019). The NLRP3 inflammasome: an overview of mechanisms of activation and regulation. Int J Mol Sci.

[24] D'Errico M, Parlanti E, Pascucci B, Filomeni G, Mastroberardino PG, Dogliotti E (2021). The interplay between mitochondrial functionality and genome integrity in the prevention of human neurologic diseases. Arch Biochem Biophys.

[25] Nicoletti V, Palermo G, Del Prete E, Mancuso M, Ceravolo R (2021). Understanding the multiple role of mitochondria in Parkinson's disease and related disorders: lesson from genetics and protein-interaction network. Front Cell Dev Biol.

[26] Heavener KS, Bradshaw EM (2022). The aging immune system in Alzheimer's and Parkinson's diseases. Semin Immunopathol.

[27] Li Y, Xia Y, Yin S, Wan F, Hu J, Kou L, Sun Y, Wu J, Zhou Q, Huang J (2021). Targeting microglial α-synuclein/TLRs/NF-kappaB/NLRP3 inflammasome axis in Parkinson's disease. Front Immunol.

[28] Heidari A, Yazdanpanah N, Rezaei N (2022). The role of Toll-like receptors and neuroinflammation in Parkinson's disease. J Neuroinflammation.

[29] Collier TJ, Kanaan NM, Kordower JH (2017). Aging and Parkinson's disease: different sides of the same coin?. Mov Disord.

[30] Goronzy JJ, Li G, Yang Z, Weyand CM (2013). The janus head of T cell aging—autoimmunity and immunodeficiency. Front Immunol.

[31] Yan Z, Yang W, Wei H, Dean MN, Standaert DG, Cutter GR, Benveniste EN, Qin H (2021). Dysregulation of the adaptive immune system in patients with early-stage Parkinson disease. Neurol Neuroimmunol Neuroinflamm.

[32] Glass CK, Saijo K, Winner B, Marchetto MC, Gage FH (2010). Mechanisms underlying inflammation in neurodegeneration. Cell.

[33] Verma DK, Seo BA, Ghosh A, Ma S-X, Hernandez-Quijada K, Andersen JK, Ko HS, Kim Y-H (2021). Alpha-synuclein preformed fibrils induce cellular senescence in Parkinson’s disease models. Cells.

[34] Santoro A, Spinelli CC, Martucciello S, Nori SL, Capunzo M, Puca AA, Ciaglia E (2018). Innate immunity and cellular senescence: the good and the bad in the developmental and aged brain. J Leukoc Biol.

[35] Calabrese V, Santoro A, Monti D, Crupi R, Di Paola R, Latteri S, Cuzzocrea S, Zappia M, Giordano J, Calabrese EJ (2018). Aging and Parkinson's Disease: inflammaging, neuroinflammation and biological remodeling as key factors in pathogenesis. Free Radic Biol Med.

[36] Chen QL, Yin HR, He QY, Wang Y (2021). Targeting the NLRP3 inflammasome as new therapeutic avenue for inflammatory bowel disease. Biomed Pharmacother.

[37] Rodrigues LP, Teixeira VR, Alencar-Silva T, Simonassi-Paiva B, Pereira RW, Pogue R, Carvalho JL (2021). Hallmarks of aging and immunosenescence: connecting the dots. Cytokine Growth Factor Rev.

[38] Hu MY, Lin YY, Zhang BJ, Lu DL, Lu ZQ, Cai W (2019). Update of inflammasome activation in microglia/macrophage in aging and aging-related disease. CNS Neurosci Ther.

[39] Gasiorowska A, Wydrych M, Drapich P, Zadrozny M, Steczkowska M, Niewiadomski W, Niewiadomska G (2021). The biology and pathobiology of glutamatergic, cholinergic, and dopaminergic signaling in the aging brain. Front Aging Neurosci.

[40] Lecours C, Bordeleau M, Cantin L, Parent M, Paolo TD, Tremblay M (2018). Microglial implication in Parkinson's disease: loss of beneficial physiological roles or gain of inflammatory functions?. Front Cell Neurosci.

[41] López-Otín C, Blasco MA, Partridge L, Serrano M, Kroemer G (2013). The hallmarks of aging. Cell.

[42] Rasheed M, Liang J, Wang C, Deng Y, Chen Z (2021). Epigenetic regulation of neuroinflammation in Parkinson's disease. Int J Mol Sci.

[43] Hashioka S, Klegeris A, Schwab C, McGeer PL (2009). Interferon-gamma-dependent cytotoxic activation of human astrocytes and astrocytoma cells. Neurobiol Aging.

[44] Neves KR, Nobre HV, Leal LK, de Andrade GM, Brito GA, Viana GS (2015). Pentoxifylline neuroprotective effects are possibly related to its anti-inflammatory and TNF-alpha inhibitory properties, in the 6-OHDA model of Parkinson's disease. Parkinsons Dis.

[45] Martin-Ruiz C, Williams-Gray CH, Yarnall AJ, Boucher JJ, Lawson RA, Wijeyekoon RS, Barker RA, Kolenda C, Parker C, Burn DJ (2020). Senescence and inflammatory markers for predicting clinical progression in Parkinson's disease: the ICICLE-PD study. J Parkinsons Dis.

[46] Roh JS, Sohn DH (2018). Damage-associated molecular patterns in inflammatory diseases. Immune Netw.

[47] Mejias NH, Martinez CC, Stephens ME, de Rivero Vaccari JP (2018). Contribution of the inflammasome to inflammaging. J Inflamm (Lond).

[48] Wang Z, Meng S, Cao L, Chen Y, Zuo Z, Peng S (2018). Critical role of NLRP3-caspase-1 pathway in age-dependent isoflurane-induced microglial inflammatory response and cognitive impairment. J Neuroinflammation.

[49] Latz E, Duewell P (2018). NLRP3 inflammasome activation in inflammaging. Semin Immunol.

[50] Cianciulli A, Porro C, Calvello R, Trotta T, Lofrumento DD, Panaro MA (2020). Microglia mediated neuroinflammation: focus on PI3K modulation. Biomolecules.

[51] Di Benedetto S, Müller L, Wenger E, Düzel S, Pawelec G (2017). Contribution of neuroinflammation and immunity to brain aging and the mitigating effects of physical and cognitive interventions. Neurosci Biobehav Rev.

[52] Gamage R, Wagnon I, Rossetti I, Childs R, Niedermayer G, Chesworth R, Gyengesi E (2020). Cholinergic modulation of glial function during aging and chronic neuroinflammation. Front Cell Neurosci.

[53] Stout-Delgado HW, Vaughan SE, Shirali AC, Jaramillo RJ, Harrod KS (2012). Impaired NLRP3 inflammasome function in elderly mice during influenza infection is rescued by treatment with nigericin. J Immunol.

[54] Youm YH, Grant RW, McCabe LR, Albarado DC, Nguyen KY, Ravussin A, Pistell P, Newman S, Carter R, Laque A (2013). Canonical Nlrp3 inflammasome links systemic low-grade inflammation to functional decline in aging. Cell Metab.

[55] Yan Y, Jiang W, Liu L, Wang X, Ding C, Tian Z, Zhou R (2015). Dopamine controls systemic inflammation through inhibition of NLRP3 inflammasome. Cell.

[56] Picca A, Guerra F, Calvani R, Romano R, Coelho-Júnior HJ, Bucci C, Marzetti E (2021). Mitochondrial dysfunction, protein misfolding and neuroinflammation in Parkinson's disease: roads to biomarker discovery. Biomolecules.

[57] Engelhardt B, Vajkoczy P, Weller RO (2017). The movers and shapers in immune privilege of the CNS. Nat Immunol.

[58] Negi N, Das BK (2018). CNS: not an immunoprivilaged site anymore but a virtual secondary lymphoid organ. Int Rev Immunol.

[59] Hornung V, Rothenfusser S, Britsch S, Krug A, Jahrsdörfer B, Giese T, Endres S, Hartmann G (2002). Quantitative expression of toll-like receptor 1–10 mRNA in cellular subsets of human peripheral blood mononuclear cells and sensitivity to CpG oligodeoxynucleotides. J Immunol.

[60] Farooq M, Batool M, Kim MS, Choi S (2021). Toll-like receptors as a therapeutic target in the era of immunotherapies. Front Cell Dev Biol.

[61] Gorecki AM, Anyaegbu CC, Anderton RS (2021). TLR2 and TLR4 in Parkinson’s disease pathogenesis: the environment takes a toll on the gut. Translational Neurodegeneration.

[62] Caputi V, Giron MC (2018). Microbiome-gut-brain axis and toll-like receptors in Parkinson's disease. Int J Mol Sci.

[63] Labzin LI, Heneka MT, Latz E (2018). Innate immunity and neurodegeneration. Annu Rev Med.

[64] Maatouk L, Compagnion AC, Sauvage MC, Bemelmans AP, Leclere-Turbant S, Cirotteau V, Tohme M, Beke A, Trichet M, Bazin V (2018). TLR9 activation via microglial glucocorticoid receptors contributes to degeneration of midbrain dopamine neurons. Nat Commun.

[65] Guo H, Callaway JB, Ting JP (2015). Inflammasomes: mechanism of action, role in disease, and therapeutics. Nat Med.

[66] Swanson KV, Deng M, Ting JPY (2019). The NLRP3 inflammasome: molecular activation and regulation to therapeutics. Nat Rev Immunol.

[67] Kinra M, Nampoothiri M, Arora D, Mudgal J (2022). Reviewing the importance of TLR-NLRP3-pyroptosis pathway and mechanism of experimental NLRP3 inflammasome inhibitors. Scand J Immunol.

[68] Pike AF, Szabò I, Veerhuis R, Bubacco L (2022). The potential convergence of NLRP3 inflammasome, potassium, and dopamine mechanisms in Parkinson’s disease. npj Parkinson's Dis.

[69] Sarkar S, Malovic E, Harishchandra DS, Ghaisas S, Panicker N, Charli A, Palanisamy BN, Rokad D, Jin H, Anantharam V (2017). Mitochondrial impairment in microglia amplifies NLRP3 inflammasome proinflammatory signaling in cell culture and animal models of Parkinson's disease. npj Parkinsons Dis.

[70] Nakahira K, Haspel JA, Rathinam VAK, Lee S-J, Dolinay T, Lam HC, Englert JA, Rabinovitch M, Cernadas M, Kim HP (2011). Autophagy proteins regulate innate immune responses by inhibiting the release of mitochondrial DNA mediated by the NALP3 inflammasome. Nat Immunol.

[71] Wang S, Yuan YH, Chen NH, Wang HB (2019). The mechanisms of NLRP3 inflammasome/pyroptosis activation and their role in Parkinson's disease. Int Immunopharmacol.

[72] Ahmed S, Kwatra M, Ranjan Panda S, Murty USN, Naidu VGM (2021). Andrographolide suppresses NLRP3 inflammasome activation in microglia through induction of parkin-mediated mitophagy in in-vitro and in-vivo models of Parkinson disease. Brain Behav Immun.

[73] Elliott EI, Miller AN, Banoth B, Iyer SS, Stotland A, Weiss JP, Gottlieb RA, Sutterwala FS, Cassel SL (2018). Cutting edge: mitochondrial assembly of the NLRP3 inflammasome complex is initiated at priming. J Immunol.

[74] Won J-H, Park S, Hong S, Son S, Yu J-W (2015). Rotenone-induced impairment of mitochondrial electron transport chain confers a selective priming signal for NLRP3 inflammasome activation*. J Biol Chem.

[75] Groß CJ, Mishra R, Schneider KS, Médard G, Wettmarshausen J, Dittlein DC, Shi H, Gorka O, Koenig PA, Fromm S (2016). K(+) Efflux-independent NLRP3 inflammasome activation by small molecules targeting mitochondria. Immunity.

[76] Prasad EM, Hung SY (2021). Current therapies in clinical trials of Parkinson's disease: a 2021 update. Pharmaceuticals (Basel).

[77] Caplan IF, Maguire-Zeiss KA (2018). Toll-like receptor 2 signaling and current approaches for therapeutic modulation in synucleinopathies. Front Pharmacol.

[78] Gambuzza ME, Sofo V, Salmeri FM, Soraci L, Marino S, Bramanti P (2014). Toll-like receptors in Alzheimer's disease: a therapeutic perspective. CNS Neurol Disord Drug Targets.

[79] Wietzorrek G, Drexel M, Trieb M, Santos-Sierra S (2019). Anti-inflammatory activity of small-molecule antagonists of Toll-like receptor 2 (TLR2) in mice. Immunobiology.

[80] Zhou Y, Chen Y, Xu C, Zhang H, Lin C (2020). TLR4 targeting as a promising therapeutic strategy for Alzheimer disease treatment. Front Neurosci.

[81] Ping Z, Xiaomu W, Xufang X, Liang S (2019). Vinpocetine regulates levels of circulating TLRs in Parkinson's disease patients. Neurol Sci.

[82] Kim C, Spencer B, Rockenstein E, Yamakado H, Mante M, Adame A, Fields JA, Masliah D, Iba M, Lee HJ (2018). Immunotherapy targeting toll-like receptor 2 alleviates neurodegeneration in models of synucleinopathy by modulating α-synuclein transmission and neuroinflammation. Mol Neurodegener.

[83] Cui B, Guo X, You Y, Fu R (2019). Farrerol attenuates MPP(+) -induced inflammatory response by TLR4 signaling in a microglia cell line. Phytother Res.

[84] Yang Y-L, Cheng X, Li W-H, Liu M, Wang Y-H, Du G-H (2019). Kaempferol attenuates LPS-induced striatum injury in mice involving anti-neuroinflammation, maintaining BBB integrity, and down-regulating the HMGB1/TLR4 pathway. Int J Mol Sci.

[85] Muhammad T, Ikram M, Ullah R, Rehman SU, Kim MO (2019). Hesperetin, a citrus flavonoid, attenuates LPS-induced neuroinflammation, apoptosis and memory impairments by modulating TLR4/NF-κB signaling. Nutrients.

[86] Daniele SG, Béraud D, Davenport C, Cheng K, Yin H, Maguire-Zeiss KA (2015). Activation of MyD88-dependent TLR1/2 signaling by misfolded α-synuclein, a protein linked to neurodegenerative disorders. Sci Signal.

[87] Zeng KW, Zhang T, Fu H, Liu GX, Wang XM (2012). Schisandrin B exerts anti-neuroinflammatory activity by inhibiting the Toll-like receptor 4-dependent MyD88/IKK/NF-κB signaling pathway in lipopolysaccharide-induced microglia. Eur J Pharmacol.

[88] Yang L, Zhou R, Tong Y, Chen P, Shen Y, Miao S, Liu X (2020). Neuroprotection by dihydrotestosterone in LPS-induced neuroinflammation. Neurobiol Dis.

[89] Haddadi R, Nayebi AM, Eyvari Brooshghalan S (2018). Silymarin prevents apoptosis through inhibiting the Bax/caspase-3 expression and suppresses toll like receptor-4 pathway in the SNc of 6-OHDA intoxicated rats. Biomed Pharmacother.

[90] Zhou J, Deng Y, Li F, Yin C, Shi J, Gong Q (2019). Icariside II attenuates lipopolysaccharide-induced neuroinflammation through inhibiting TLR4/MyD88/NF-κB pathway in rats. Biomed Pharmacother.

[91] Sun MF, Zhu YL, Zhou ZL, Jia XB, Xu YD, Yang Q, Cui C, Shen YQ (2018). Neuroprotective effects of fecal microbiota transplantation on MPTP-induced Parkinson's disease mice: Gut microbiota, glial reaction and TLR4/TNF-α signaling pathway. Brain Behav Immun.

[92] Zhao Z, Ning J, Bao X-Q, Shang M, Ma J, Li G, Zhang D (2021). Fecal microbiota transplantation protects rotenone-induced Parkinson’s disease mice via suppressing inflammation mediated by the lipopolysaccharide-TLR4 signaling pathway through the microbiota-gut-brain axis. Microbiome.

[93] Xue L-J, Yang X-Z, Tong Q, Shen P, Ma S-J, Wu S-N, Zheng J-L, Wang H-G (2020). Fecal microbiota transplantation therapy for Parkinson's disease: a preliminary study. Medicine.

[94] Segal A, Zlotnik Y, Moyal-Atias K, Abuhasira R, Ifergane G (2021). Fecal microbiota transplant as a potential treatment for Parkinson's disease—a case series. Clin Neurol Neurosurg.

[95] Gordon R, Albornoz EA, Christie DC, Langley MR, Kumar V, Mantovani S, Robertson AAB, Butler MS, Rowe DB, O'Neill LA (2018). Inflammasome inhibition prevents α-synuclein pathology and dopaminergic neurodegeneration in mice. Sci Transl Med.

[96] Pan X, Liu X, Zhao H, Wu B, Liu G (2020). Antioxidant, anti-inflammatory and neuroprotective effect of kaempferol on rotenone-induced Parkinson’s disease model of rats and SH-S5Y5 cells by preventing loss of tyrosine hydroxylase. J Funct Foods.

[97] Lin K-H, Li C-Y, Hsu Y-M, Tsai C-H, Tsai F-J, Tang C-H, Yang J-S, Wang Z-H, Yin M-C (2019). Oridonin, a natural diterpenoid, protected NGF-differentiated PC12 cells against MPP+- and kainic acid-induced injury. Food Chem Toxicol.

[98] Ren M, Han M, Wei X, Guo Y, Shi H, Zhang X, Perez RG, Lou H (2017). FTY720 attenuates 6-OHDA-associated dopaminergic degeneration in cellular and mouse Parkinsonian models. Neurochem Res.

[99] Territo PR, Zarrinmayeh H (2021). P2X(7) receptors in neurodegeneration: potential therapeutic applications from basic to clinical approaches. Front Cell Neurosci.

[100] Lin MW, Lin CC, Chen YH, Yang HB, Hung SY (2019). Celastrol inhibits dopaminergic neuronal death of Parkinson's disease through activating mitophagy. Antioxidants (Basel)..

[101] Arab HH, Safar MM, Shahin NN (2021). Targeting ROS-dependent AKT/GSK-3β/NF-κB and DJ-1/Nrf2 pathways by dapagliflozin attenuates neuronal injury and motor dysfunction in rotenone-induced Parkinson's disease rat model. ACS Chem Neurosci.

[102] Valera E, Mante M, Anderson S, Rockenstein E, Masliah E (2015). Lenalidomide reduces microglial activation and behavioral deficits in a transgenic model of Parkinson’s disease. J Neuroinflammation.

[103] Cankara FN, Günaydın C, Bilge SS, Özmen Ö, Kortholt A (2020). The neuroprotective action of lenalidomide on rotenone model of Parkinson's disease: neurotrophic and supportive actions in the substantia nigra pars compacta. Neurosci Lett.

[104] Huang YY, Zhang Q, Zhang JN, Zhang YN, Gu L, Yang HM, Xia N, Wang XM, Zhang H (2018). Triptolide up-regulates metabotropic glutamate receptor 5 to inhibit microglia activation in the lipopolysaccharide-induced model of Parkinson's disease. Brain Behav Immun.

[105] Zhang FX, Xu RS (2018). Juglanin ameliorates LPS-induced neuroinflammation in animal models of Parkinson's disease and cell culture via inactivating TLR4/NF-κB pathway. Biomed Pharmacother.

[106] Pan Q, Ban Y, Khan S (2021). Antioxidant activity of calycosin against α-synuclein amyloid fibrils-induced oxidative stress in neural-like cells as a model of preventive care studies in Parkinson's disease. Int J Biol Macromol.

[107] Chaouhan HS, Li X, Sun K-T, Wang IK, Yu T-M, Yu S-H, Chen K-B, Lin W-Y, Li C-Y (2022). Calycosin alleviates paraquat-induced neurodegeneration by improving mitochondrial functions and regulating autophagy in a drosophila model of Parkinson's disease. Antioxidants (Basel, Switzerland).

[108] Lee SL, Tu SC, Hsu MY, Chin TY (2021). Diosgenin prevents microglial activation and protects dopaminergic neurons from lipopolysaccharide-induced neural damage in vitro and in vivo. Int J Mol Sci.

[109] Jing H, Wang S, Wang M, Fu W, Zhang C, Xu D (2017). Isobavachalcone attenuates MPTP-induced Parkinson's disease in mice by inhibition of microglial activation through NF-κB pathway. PLoS ONE.

[110] Hou L, Sun F, Huang R, Sun W, Zhang D, Wang Q (2019). Inhibition of NADPH oxidase by apocynin prevents learning and memory deficits in a mouse Parkinson's disease model. Redox Biol.

[111] Wang Q, Qian L, Chen SH, Chu CH, Wilson B, Oyarzabal E, Ali S, Robinson B, Rao D, Hong JS (2015). Post-treatment with an ultra-low dose of NADPH oxidase inhibitor diphenyleneiodonium attenuates disease progression in multiple Parkinson's disease models. Brain.

[112] Proukakis C, Dudzik CG, Brier T, MacKay DS, Cooper JM, Millhauser GL, Houlden H, Schapira AH (2013). A novel α-synuclein missense mutation in Parkinson disease. Neurology.

[113] Li D, Yang H, Ma J, Luo S, Chen S, Gu Q (2018). MicroRNA-30e regulates neuroinflammation in MPTP model of Parkinson's disease by targeting Nlrp3. Hum Cell.

[114] Wu JR, Wang J, Zhou SK, Yang L, Yin JL, Cao JP, Cheng YB (2015). Necrostatin-1 protection of dopaminergic neurons. Neural Regen Res.

[115] Wu L, Xu Q, Zhou M, Chen Y, Jiang C, Jiang Y, Lin Y, He Q, Zhao L, Dong Y (2022). Plasma miR-153 and miR-223 levels as potential biomarkers in Parkinson’s disease. Front Neurosci.

[116] Deng W, Yang Z, Yue H, Ou Y, Hu W, Sun P (2020). Disulfiram suppresses NLRP3 inflammasome activation to treat peritoneal and gouty inflammation. Free Radic Biol Med.

[117] Zhao Y, Guo Q, Zhu Q, Tan R, Bai D, Bu X, Lin B, Zhao K, Pan C, Chen H (2019). Flavonoid VI-16 protects against DSS-induced colitis by inhibiting Txnip-dependent NLRP3 inflammasome activation in macrophages via reducing oxidative stress. Mucosal Immunol.

[118] Olajide OA, Sarker SD (2020). Alzheimer's disease: natural products as inhibitors of neuroinflammation. Inflammopharmacology.

[119] Tansey MG, Wallings RL, Houser MC, Herrick MK, Keating CE, Joers V (2022). Inflammation and immune dysfunction in Parkinson disease. Nat Rev Immunol.

[120] Hoy SM (2019). Levodopa/carbidopa enteral suspension: a review in advanced Parkinson's disease. Drugs.

[121] Hu C, Ke CJ, Wu C (2020). Identification of biomarkers for early diagnosis of Parkinson's disease by multi-omics joint analysis. Saudi J Biol Sci.

[122] Bellettato CM, Scarpa M (2018). Possible strategies to cross the blood–brain barrier. Ital J Pediatr.

[123] Ohta S, Kikuchi E, Ishijima A, Azuma T, Sakuma I, Ito T (2020). Investigating the optimum size of nanoparticles for their delivery into the brain assisted by focused ultrasound-induced blood-brain barrier opening. Sci Rep.

[124] Gouda NA, Elkamhawy A, Cho J (2022). Emerging therapeutic strategies for Parkinson's disease and future prospects: a 2021 update. Biomedicines.

[125] Chatterjee D, Bhatt M, Butler D, De Genst E, Dobson CM, Messer A, Kordower JH (2018). Proteasome-targeted nanobodies alleviate pathology and functional decline in an α-synuclein-based Parkinson's disease model. NPJ Parkinsons Dis.

[126] Feng X, Liu J, Xu W, Li G, Ding J (2020). Tackling autoimmunity with nanomedicines. Nanomedicine (Lond).

[127] Chen X, Zhou Y, Yu J (2019). Exosome-like nanoparticles from ginger rhizomes inhibited NLRP3 inflammasome activation. Mol Pharm.

[128] Cao M, Cai L (2022). Nanoparticle emulsions enhance the inhibition of NLRP3. Int J Mol Sci.

[129] Liu B, Li X, Yu H, Shi X, Zhou Y, Alvarez S, Naldrett MJ, Kachman SD, Ro S-H, Sun X (2021). Therapeutic potential of garlic chive-derived vesicle-like nanoparticles in NLRP3 inflammasome-mediated inflammatory diseases. Theranostics.

[130] Zeb A, Rana I, Choi HI, Lee CH, Baek SW, Lim CW, Khan N, Arif ST, Sahar NU, Alvi AM (2020). Potential and applications of nanocarriers for efficient delivery of biopharmaceuticals. Pharmaceutics.

